# Postobstructive Pulmonary Edema following Tonsillectomy/Adenoidectomy in a 2-Year-Old with Poland-Moebius Syndrome

**DOI:** 10.1155/2016/5431809

**Published:** 2016-01-28

**Authors:** Tanisha Powell, Nirupma Sharma, Kathleen T. McKie

**Affiliations:** Department of Pediatrics, Augusta University (Formerly Georgia Regents University), Augusta, GA 30912, USA

## Abstract

A 2-year-old male with Poland-Moebius syndrome was transferred from a local hospital to the Pediatric ICU at Children's Hospital of Georgia for suspected postobstructive pulmonary edema (POPE) after tonsillectomy/adenoidectomy (T&A). The patient's respiratory status ultimately declined and he developed respiratory failure. Imaging suggested pulmonary edema as well as a left-sided pneumonia. Echocardiogram showed pulmonary hypertension and airway exam via direct fiberoptic bronchoscopy revealed tracheomalacia and bronchomalacia. He developed acute respiratory distress syndrome (ARDS) and remained intubated for ten days. This case highlights the association between congenital upper body abnormalities with cranial nerve dysfunction and the development of POPE with delayed resolution of symptoms. Patients with upper body abnormalities as above are at great risk of postoperative complications and should therefore be managed in a tertiary-care facility.

## 1. Introduction

We present a case of postobstructive pulmonary edema after T&A in a child with Poland-Moebius syndrome. These relatively uncommon anomalies likely contributed to postoperative complications for this child including POPE and ARDS. Patients with similar upper body abnormalities should be recognized as high risk of development of postoperative complications following T&A.

## 2. Case Report

A 2-year-old male with Poland-Moebius syndrome had tonsillectomy/adenoidectomy (T&A) performed at a local general hospital due to snoring and apnea-hypopnea index (AHI) of 10–15 on multiple prior polysomnograms (normal AHI in children is <1). Details of his anesthetic management during surgery are not available; however no immediate perioperative complications such as laryngospasm or retained pack were reported. While in recovery, he exhibited increased work of breathing, coarse breath sounds, and lethargy. He was admitted to an adult ICU and treated with IV diuretics and oxygen with continuous positive airway pressure (CPAP). He improved briefly and CPAP was stopped, but he had an episode of vomiting after administration of oral Percocet and oxygen saturation acutely dropped to the upper 60s. CPAP was restarted and saturation increased to 90%. A chest X-ray showed peribronchial and perihilar interstitial thickening and known left lateral rib deformities. Due to unstable respiratory status, he was transferred to the Pediatric ICU at the Children's Hospital of Georgia within 12 hours of surgery.

On admission to our PICU he was noted to be in moderate respiratory distress with respiratory rate 56; subcostal and intercostal retractions were present. The patient's clinical picture was concerning for postobstructive pulmonary edema (POPE) although the differential diagnosis also included aspiration pneumonia. Repeat chest X-ray showed patchy infiltrates consistent with pulmonary edema. He was started on high-flow oxygen. Diuretic therapy was initiated and intravenous fluids were restricted. Pulmonary toilet was maintained with hypertonic saline and chest physiotherapy. IV dexamethasone was given to decrease airway swelling. He was noted to have poor swallowing of secretions, and a modified barium swallow done later in the course of his hospital stay confirmed dysphagia. On day 5, he developed fever and was treated empirically with ceftriaxone for pneumonia. His respiratory status continued to decline and he ultimately developed respiratory failure, requiring intubation on day 7 of hospital stay. Ventilator settings included respiratory rate 30, PEEP +9, and PC 27 with FiO_2_ 80% to maintain sats in the 90s. Repeat chest X-rays and chest CT showed persistent, diffuse opacities. Clinical picture suggested acute respiratory distress syndrome (ARDS) and diuretic therapy was started for fluid management. Tracheal aspirate grew methicillin-susceptible staph aureus (MSSA). Direct fiberoptic bronchoscopy done on day 8 showed significant tracheomalacia and bronchomalacia. An echocardiogram on day 10 showed mildly dilated right ventricle and elevated right ventricular systolic pressure at 3/4 systemic pressure consistent with a diagnosis of pulmonary hypertension; these findings differed significantly from previous normal echocardiogram done at 14 months of age. Sildenafil was started for treatment of pulmonary hypertension and subsequent echocardiograms showed improvement in right ventricular systolic pressure. He remained intubated for ten days but eventually was extubated to noninvasive ventilatory support with BiPAP. He was weaned to room air with continued use of BiPaP at night and later discharged to a rehabilitation facility after a total inpatient stay of 30 days.

## 3. Discussion

We present a case of POPE Type II after T&A in a child with Poland-Moebius syndrome. POPE (or negative pressure pulmonary edema) is a potentially fatal complication typically seen after relief of upper airway obstruction. POPE Type I develops immediately after relief of an acute airway obstruction whereas POPE Type II typically occurs after relief of chronic obstruction (as seen with obstructive sleep apnea caused by adenotonsillar hypertrophy). Patients with chronic obstruction appear to exhibit a level of autopositive end expiratory pressure (PEEP) with a concomitant increase in end expiratory lung volume. When the obstruction is relieved, lung volumes and pressures return to normal, creating a negative intrapulmonary pressure which may result in interstitial fluid transudation and pulmonary edema [1]. POPE usually presents immediately after extubation, but sometimes the onset is delayed up to a few hours in the postoperative period. Patients present with tachypnea, desaturation, frothy secretions, and rales. Chest radiographs show bilateral interstitial infiltrates. Treatment involves positive pressure ventilation, oxygen, restriction of fluids, and diuretic therapy [2].

In addition to standard risk factors for post-T&A complications, including young age and AHI >5, our patient's Poland syndrome (in the absence of left-sided pectoralis muscles and hypoplasia of ribs 3–5) and Moebius syndrome (affecting his right-sided cranial nerves) undoubtedly impacted his clinical course. Poland syndrome occurs in 1 in 20,000 live births; Poland syndrome combined with Moebius syndrome has an estimated prevalence of 1 : 500,000 [3]. Poland syndrome has been associated with decreased pulmonary function related to absence of respiratory muscles or decreased muscle function. Patients with Poland syndrome may exhibit paradoxical respirations due to herniation of a portion of the lung through the defect, causing inadequate ventilation and making need for long-term, postoperative ventilatory support more likely [4]. Delayed clearance of secretions from inadequate pulmonary toilet can cause complications such as pneumonia. Acute respiratory failure can lead to pulmonary hypertension with elevated right ventricular pressure. In Moebius syndrome, tracheomalacia, palatal weakness, and uvular weakness can cause difficulty with intubation and/or loss of the airway. Difficulty swallowing and eliminating oral secretions can lead to aspiration and chronic pulmonary disease [5, 6]. The combination of swallowing dysfunction with inability to clear secretions along with weakness of the airway likely caused worsening of this patient's respiratory status and delayed resolution of his illness.

This case highlights the association between congenital upper body abnormalities with cranial nerve defects (as in Poland-Moebius syndrome) and the development of POPE with delayed resolution. This should be anticipated following surgery in any child with similar risk factors. These patients should have surgical procedures performed in a tertiary-care facility with admission to a Pediatric ICU postoperatively for optimal management of these complications.
